# Flow cytometry analysis of the subpopulations of mouse keratinocytes and skin immune cells

**DOI:** 10.1016/j.xpro.2021.101052

**Published:** 2021-12-16

**Authors:** Keiko Sakamoto, Shubham Goel, Atsuko Funakoshi, Tetsuya Honda, Keisuke Nagao

**Affiliations:** 1 Cutaneous Leukocyte Biology Section, Dermatology Branch, National Institute of Arthritis and Musculoskeletal and Skin Diseases, National Institutes of Health, Bethesda, MD 20892, USA; 2 Department of Dermatology, Hamamatsu University School of Medicine, Hamamatsu, Shizuoka 431-3192, Japan

**Keywords:** Cell isolation, Flow Cytometry/Mass Cytometry, Immunology, Model Organisms

## Abstract

Skin is our body’s outermost physical barrier and an immunological interface enriched with various immune and non-immune cells. However, efficient generation of single-cell suspensions for flow cytometry analysis can be challenging. Here, we provide protocols to obtain epidermal and whole skin cell suspensions as well as gating strategies to identify mouse keratinocytes and skin immune cell subsets via flow cytometry.

For complete details on the use and execution of this protocol, please refer to Sakamoto et al. (2021).

## Highlights

• Preparation of single-cell suspension from mouse epidermis and whole skin
• Antibody staining for keratinocytes, ILCs, and myeloid cells for flow cytometry
• Gating strategy for keratinocyte, ILC, and myeloid cell subsets

## Graphical abstract

**Figure:**
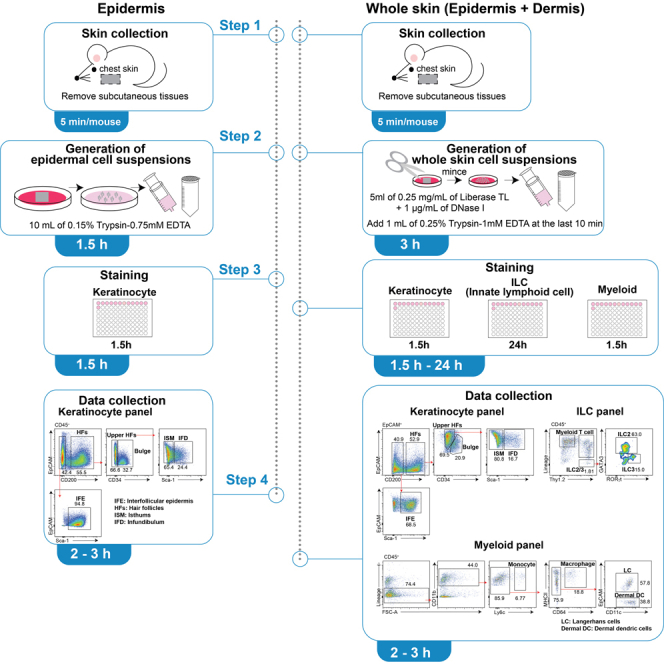

## Before you begin

Mice were bred and/or maintained in the specific pathogen-free facility in accordance with the Guide for the Care and Use of Laboratory Animals. All experiments were performed at National Institute of Arthritis and Musculoskeletal Skin Diseases under an animal study proposal approved by the NIAMS Animal Care and Use Committee.

### Mice

Before working with this protocol, secure sufficient numbers of age matched female C57BL/6 mice for each experiment. We generally use only female mice because adult male mouse skin is relatively thick and cannot be efficiently digested (Azzi et al., 2005). Male mice also tend to have traumatic skin wounds that lead to increased inflammatory cell infiltrates (Kashem and Kaplan, 2018). One might start harvesting 3 to 5 mice at a time and then increase the numbers of mice per harvest after becoming competent in all experimental procedures. Use exactly age-matched mice for control and experimental groups because hair follicle (HF) and immune cell numbers can be affected by the age of mice and the hair cycle, which undergoes bouts of hair growth and regression in an age-dependent manner (Schneider et al., 2009).

### Antibody panel preparation

**Timing: 0.5–1 h**

1. Prepare the flow cytometry antibody panel for keratinocyte, innate lymphoid cell (ILC), and myeloid cell (Tables 1, 2, 3, and 4). Make sure that you have enough antibodies for the total number of samples you want to analyze, which should be confirmed on the prior day. Make an antibody master mix immediately before the staining step. Use a 5% FACS buffer for dilution.

**Table 1:** Keratinocyte panel (epidermis)

Fluorophore	Marker	Clone	Final dilution
BV421	CD34	RAM34	1/100
BV711	MHC ll	M5/114.15.2	1/2000
APC-Cy7	Sca-1	D7	1/200
BUV395	CD45	30-F11	1/200
PE	CD200	OX-90	1/100
PE-Cy7	EpCAM	G8.8	1/200

**Table 2:** Keratinocyte panel (whole skin)

Fluorophore	Marker	Clone	Final dilution
BV421	CD34	RAM34	1/100
BV711	MHC ll	M5/114.15.2	1/2000
APC	CD31	MEC13.3	1/200
APC-Cy7	Sca-1	D7	1/200
BUV395	CD45	30-F11	1/200
PE	CD200	OX-90	1/100
PE-Cy7	EpCAM	G8.8	1/200

**Table 3:** Innate lymphoid cell (ILC) panel

Fluorophore	Marker	Clone	Final dilution
PerPCy5.5	CD3ε	145-2C11	1/200
	CD5	53-7.3	1/200
	CD19	6D5	1/200
	CD11b	M1/70	1/200
	CD11c	N418	1/200
	FcεRIα	MAR-1	1/200
	NK1.1	PK136	1/200
	CD2	RM2-5	1/200
BV421	CD45	30F-11	1/100
BV711	MHC ll	M5/114.15.2	1/3000
APC-Cy7	Sca-1	D7	1/200
BUV395	Thy1.2	53-2.1	1/200
PE-Cy7	CCR6	29-2L17	1/200

To detect transcription factors (GATA3 and RORγt), staining is performed after cell surface staining (Table 5).

**Table 4:** Myeloid cell panel

Fluorophore	Marker	Clone	Final dilution
PerPCy5.5	CD3ε	145-2C11	1/300
	CD19	6D5	1/300
	NK1.1	PK136	1/300
	Siglec-F	E50-2440	1/300
BV421	CD11b	30F-11	1/400
BV650	CD11c	N418	1/200
BV711	Ly6c	HK1.4	1/400
APC-Cy7	EpCAM	G8.8	1/200
BUV395	CD45	30-F11	1/200
PE	CCR2	475301	1/200
PE-CF594	CD64	X54-5/7.1	1/200
PE-Cy7	MHC ll	M5/114.15.2	1/5000

## Key resources table

**Table:** 

REAGENT or RESOURCE	SOURCE	IDENTIFIER
**Antibodies**		

BV421 anti-mouse CD34, clone RAM34 (1/100)	BD Horizon	Cat# 562608; RRID: AB_11154576
BV711 anti-mouse IA-IE, clone M5/114.15.2 (keratinocyte panel: 1/2000, ILC panel: 1/3000)	BD Horizon	Cat# 563414; RRID: AB_2738191
AF647 anti-mouse CD31, clone MEC13.3 (1/200)	BioLegend	Cat# 102516; RRID: AB_2161029
APC/Cy7 anti-mouse Ly-6A/E (Sca-1), clone D7 (1/200)	BioLegend	Cat# 108126; RRID: AB_10645327
BUV395 anti-mouse CD45, clone 30-F11 (1/200)	BD Horizon	Cat# 564279; RRID: AB_2651134
PE anti-mouse CD200 (OX2), clone OX-90 (1/200)	BioLegend	Cat# 123808; RRID: AB_2073942
PE/Cy7 anti-mouse CD326 (Ep-CAM), clone G8.8 (1/200)	BioLegend	Cat# 118216; RRID: AB_1236471099
BV421 anti-mouse CD45, clone 30F-11 (1/100)	BioLegend	Cat# 103134; RRID: AB_2562559
PerCP/Cy5.5 anti-mouse CD2, clone RM2-5 (1/200)	BioLegend	Cat# 100116; RRID: AB_2563502
PerCP/Cy5.5 anti-mouse CD11c, clone N418 (1/200)	BioLegend	Cat# 117328; RRID: AB_2129641
PerCP/Cy5.5 anti-mouse CD19, clone 6D5 (ILC panel: 1/200, myeloid panel: 1/300)	BioLegend	Cat# 115534; RRID: AB_2072925
PerCP/Cy5.5 anti-mouse CD3e, clone 145-2C11 (ILC panel: 1/200, myeloid panel: 1/300)	BioLegend	Cat# 100328; RRID: AB_893318
PerCP/Cy5.5 anti-mouse CD11b, clone M1/70 (1/200)	BioLegend	Cat# 101228; RRID: AB_893232
PerCP/Cy5.5 anti-mouse CD5, clone 53-7.3 (1/200)	BioLegend	Cat# 100624; RRID: AB_2563433
PerCP/Cy5.5 anti-mouse FcεRIα, clone MAR-1 (1/200)	BioLegend	Cat# 134320; RRID: AB_10641135
PerCP/Cy5.5 anti-mouse NK-1.1, clone PK136 (ILC panel: 1/200, myeloid panel: 1/300)	BioLegend	Cat# 108728; RRID: AB_2132705
PerCP/Cy5.5 Siglec-F, clone E50-2440 (1/300)	BD Biosciences	Cat# 565526; RRID: AB_2739281
BUV395 anti-mouse Thy1.2 (CD90.2), clone 53-2.1 (1/200)	BD Horizon	Cat# 565257; RRID: AB_2739136
PE/Cy7 anti-mouse CD196 (CCR6), clone 29-2L17 (1/200)	BioLegend	Cat# 129816; RRID: AB_2072798
AF647 anti-mouse GATA3, clone L50-823 (1/20)	BD Pharmingen	Cat# 560068; RRID: AB_1645316
PE anti-mouse RORγt, clone Q31-378 (1/50)	BD Pharmingen	Cat# 562607; RRID: AB_11153137
BV421 anti-mouse CD11b, clone M1/70 (1/200)	BioLegend	Cat# 101236; RRID: AB_11203704
BV650 anti-mouse CD11c, clone N418 (1/200)	BioLegend	Cat# 117339; RRID: AB_2562414
BV711 anti-mouse Ly6c, clone HK1.4 (1/200)	BioLegend	Cat# 128037; RRID: AB_2562630
APC/Cy7 anti-mouse CD326 (Ep-CAM), clone G8.8 (1/200)	BioLegend	Cat# 118218; RRID: AB_2098648
PE-CF594 anti-mouse CD64, clone X54-5/7.1 (1/200)	BioLegend	Cat# 139320; RRID: AB_2566559
PE/Cy7 anti-mouse I-A/I-E, clone M5/114.15.2 (1/5000)	BioLegend	Cat# 107630; RRID: AB_2069376
TruStain FcX™ (anti-mouse CD16/32) Antibody, clone 93 (1/200)	BioLegend	Cat# 101320; RRID: AB_1574975
Anti-Rat and Anti-Hamster Ig κ /Negative Control Compensation Particles Set	BD Biosciences	Cat# 552845; RRID: AB_10058522

**Chemicals, peptides, and recombinant proteins**		

Trypsin-EDTA (0.05%)	GIBCO	Cat# 25300054
Trypsin-EDTA (0.25%)	GIBCO	Cat# 25200056
PBS, pH 7.4	GIBCO	Cat# 10010023
RPMI 1640 Medium	GIBCO	Cat# 11875-093
BenchMark™ Fetal Bovine Serum (FBS)	BenchMARK^TM^	Cat# 100-106
Liberase T-Flex Research Grade (500 MG)	ROCHE	Cat# 05989132001
Deoxyribonuclease I from bovine pancreas	Sigma-Aldrich	Cat# DN25-1G

**Critical commercial assays**		

Zombie Aqua Fixable Viability Kit	BioLegend	Cat# 423101
FluoroFix™ Buffer	BioLegend	Cat# 422101
eBioscience™ Foxp3/Transcription Factor Staining Buffer Set	Thermo Fisher Scientific	Cat# 00-5523-00

**Experimental models: organisms/strains**		

Mouse: C57BL/6J, female, 8–12 weeks old	The Jackson Laboratory	Jax: 000664

**Software and algorithms**		

FlowJo	FlowJo, LLC	https://www.flowjo.com/solutions/flowjo

**Other**		

Falcon® 40 μm Cell Strainer, Blue, Sterile, Individually Packaged, 50/Case	Corning	Cat# 352340
Falcon® 100 μm Cell Strainer, Yellow, Sterile, Individually Packaged, 50/Case	Corning	Cat# 352360
Falcon™ 96-Well, Non-Treated, V-Shaped-Bottom Microplate	Fisher Scientific	Cat# 08-772-212
BD® LSR II Flow Cytometer	BD Biosciences	N/A
BD® LSR Fortessa Flow Cytometer	BD Biosciences	N/A

## Materials and equipment

### 5% FACS buffer

To make PBS containing 5% fetal bovine serum, add 25 mL Fetal Bovine Serum (FBS) into 475 mL 1× PBS. Keep refrigerated or on ice.

### 40× Liberase T-Flex stock

Dissolve Liberase T-Flex Research Grade (500 mg Collagenase blend and 30 mg Thermolysin) in 1× PBS to make a 1 mg/mL Liberase stock. Store at −20°C in 1 mL aliquots for up to 6 months.

### 1× DNase stock

Dissolve 1 mg Deoxyribonuclease I from bovine pancreas in 1 mL of 0.15 M NaCl to make a 1 mg/mL DNase stock. Store at −20°C in 40 μL aliquots for up to 1 month.

### Epidermis digestion solution

Mix 5 mL of 0.25% Trypsin-1 mM EDTA and 5 mL of 0.05% Trypsin-0.5 mM EDTA to make 0.15% trypsin and 0.75 mM EDTA. This solution should be prepared on the day of sample processing and put on ice until use. Use 10 mL for each skin sample.

### Whole skin digestion solution

Dilute Liberase stock to a final concentration of 0.25 mg/mL and DNase stock to a final concentration of 1 μg/mL in RPMI (not supplemented with antibiotics, amino acids or FBS). This solution should be prepared on the day of sample processing and put on ice until use. Use 5 mL for each skin sample.

### 1× Foxp3 fixation/permeabilization working solution

Mix 1 part of Foxp3 Fixation/Permeabilization Concentrate with 3 parts of Foxp3 Fixation/Permeabilization Diluent. For example, mix 1 mL Foxp3 Fixation/Permeabilization Concentrate with 3 mL Foxp3 Fixation/Permeabilization Diluent. This solution should be prepared on the day of sample processing and stored at room temperature until use. Both concentrate and diluent are included in the Bioscience™ Foxp3/Transcription Factor Staining Buffer Set.

### 1× permeabilization buffer working solution

Mix 1 part of 10× Permeabilization Buffer with 9 parts of distilled water. For example, mix 5 mL 10× Permeabilization Buffer with 45 mL distilled water. This solution should be prepared on the day of sample processing and stored at room temperature until use. Permeabilization buffer is included in the Bioscience™ Foxp3/Transcription Factor Staining Buffer Set.

## Step-by-step method details

### Skin collection

**Timing: 10 min/mouse**

**Figure 1:**
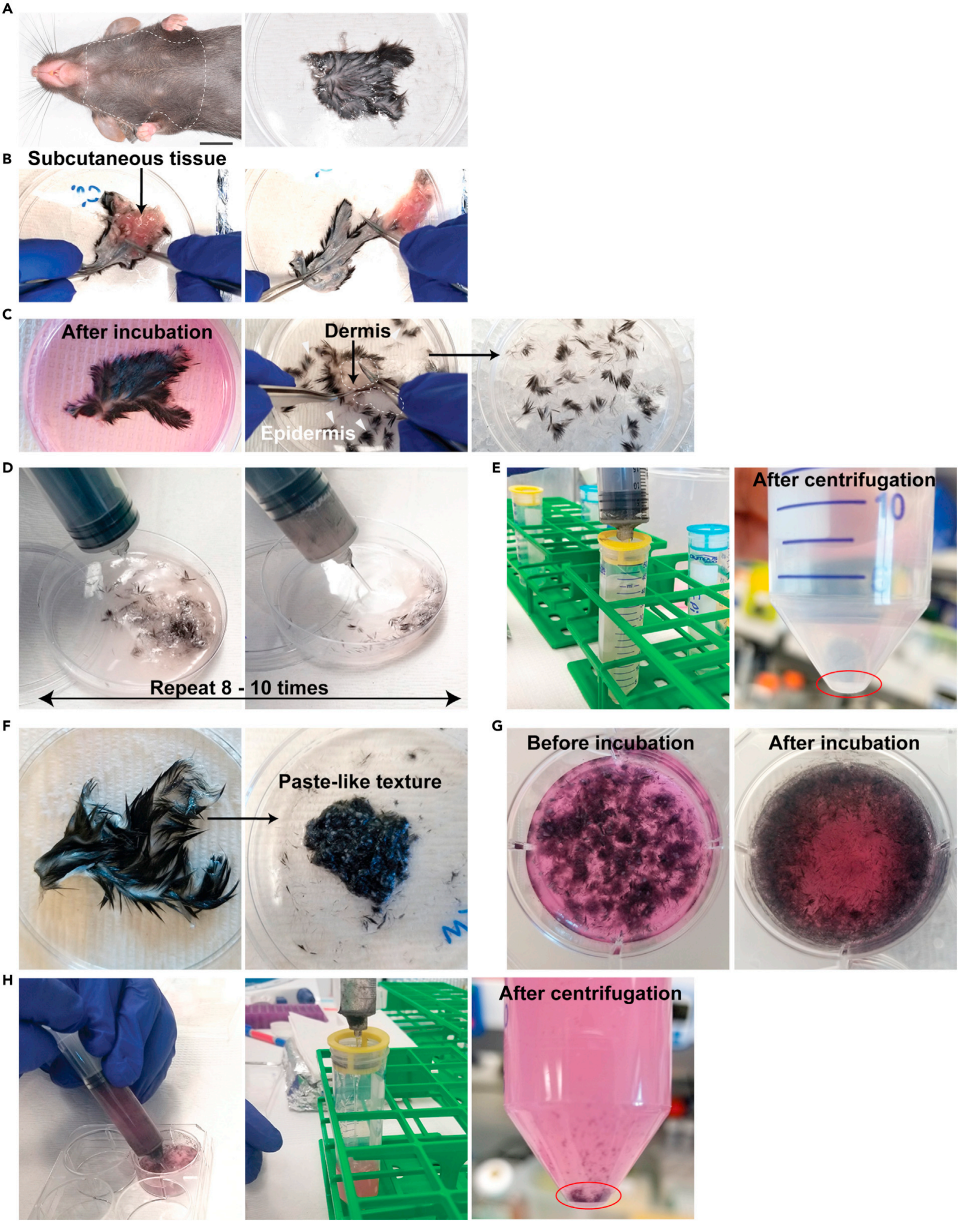
Processing the tissues (A) Harvest skin along the dotted circle. Scale bar = 1 cm. (B) Flip the skin sample surface-side down. Fix the sample by pressing down on the neck side of the sample with a pair of curved forceps. Scrape off subcutaneous tissue with another curved forceps held in your other hand. The subcutaneous tissue will come off easier when it is scraped off in the direction of the hair pattern. (C) After 45 min incubation with epidermal digestion solution, fix the neck side of the skin with a pair of curved forceps. With another curved forceps, scrape on the skin surface, following the hair direction, to detach the epidermis. Dotted circles depict the dermis. Arrowheads depict detached epidermis. (D) Pump 8 to 10 times with a 50 mL syringe. Use the lid of the petri dish to make a slope. (E) Red circle indicates cell pellet after the cell suspension is spun down. (F) Mince skin well until it gets paste-like texture using ophthalmic scissors in 35 mm petri dishes on ice. (G) Images of tissues before and after enzymatic digestion. (H) Pump 8 to 10 times with a 12 mL syringe. Red circle indicates the cell pellet after the cell suspension is spun down.
  c. If more than 5 mice need to be harvested, repeat step 1–2b.
  d. Transfer skin samples (one sample at a time) surface-side down onto the lid of petri dishes and scrape off subcutaneous tissue using two forceps (Figure 1B, Method video S1).
  e. Transfer back the skin into each petri dish and float on PBS.

1. Euthanize mice (up to 5 mice) by CO_2_ inhalation.
2. Collect skin samples from the chest area immediately after euthanization.
  a. Cut off an approximately 3 cm × 4 cm area from mouse chest skin using ophthalmic scissors. The chest area does not require prior shaving.
  b. Float each skin sample in a petri dish containing 10 mL of PBS on ice while other samples are being processed (Figure 1A).

Method video S1. Subcutaneous tissue removal, related to step 2d

### Generating cell suspension

#### Generating epidermal cell suspensions

**Timing: 1.5 h**

3. Float skin surface-side up on 10 mL of epidermis digestion solution (0.15% trypsin and 0.75 mM EDTA) in 100 mm × 15 mm petri dish. Remove any air bubbles under the skin samples using two curved forceps.
4. Incubate for 45 min at 37°C in a cell culture incubator. CO_2_ is not mandatory.
5. Briefly transfer the skin onto the lid of the petri dish and discard epidermis digestion solution by decanting. Put the skin back onto the petri dish and pour 20 mL of 4°C 5% FACS buffer into each petri dish.
6. Scrape off the epidermis from the dermis gently with two curved forceps. If enzymatic digestion is effective, the epidermal component should detach without exerting excessive force. Discard dermis (Figure 1C, Method video S2).
7. The epidermal cells are further mechanically dissociated with a 50 mL syringe (Covidien). Pump 8 to 10 times without applying too much pressure. Applying excessive force may affect cell viability (Figure 1D).
8. Filter the cell suspensions through sterile 100 μm Falcon® Cell Strainers (Corning) placed on 50 mL conical tubes. Centrifuge at 400 × *g* for 5 min at 4°C and remove the supernatant.
9. Break the cell pellets and resuspend in 10 mL of 5% FACS buffer.
10. Filter the solution through sterile 40 μm Falcon® Cell Strainers (Corning) placed on new 50 mL conical tubes.
11. Centrifuge at 400 × *g* for 5 min at 4°C. Remove the supernatant and resuspend with 300 μL of 5% FACS buffer (Figure 1E).

Method video S2. Detaching epidermis, related to step 6

#### Generating whole skin cell suspension

**Timing: 3 h**

12. Add 5 mL of whole skin digestion solution into each well of 6-well plates. Keep on ice.
13. Place the skin sample on Kim wipe very briefly to remove excess PBS.
14. Transfer each skin sample into 35 mm petri dishes and mince well using ophthalmic scissors.
15. Transfer 1 mL of whole skin digestion solution from the 6-well plate and add it to the minced tissue. Further mince until paste-like textures are obtained (Figure 1F). Mincing process should be done on ice.
16. Pour the minced skin into 6-well plates with a whole skin digestion solution.
17. Incubate for 2 h at 37°C in a cell culture incubator. CO_2_ is not mandatory.
18. Add 1 mL of 0.25% Trypsin-1 mM EDTA into each well for the last 10 min of incubation (Figure 1G).
19. Deactivate the enzymatic reaction by adding 4 mL of 4°C 5% FACS buffer into each well.
20. Mechanically dissociate with a 12 mL syringe. Pump 8 to 10 times. Excessive force should not be applied.
21. Filter the cell suspension through sterile 100 μm Falcon® Cell Strainers (Corning) placed on 50 mL conical tubes. Centrifuge at 400 × *g* for 8 min at 4°C and remove the supernatant (Figure 1H). In this final centrifugation step for whole skin, we recommend 8 min instead of 5 min to avoid cell loss since pellets may be loose compared to those obtained from epidermal cell suspensions.
22. Break the cell pellets and resuspend in 10 mL of 5% FACS buffer.
23. Filter again with 40 μm cell strainers placed on new 50 mL conical tubes.
24. Centrifuge at 400 × *g* for 8 min at 4°C. Remove supernatant and resuspend with 300 μL of 5% FACS buffer.

### Staining

**Timing: 1.5 h**

25. Transfer 150 μL of cell suspension into a 96 v-bottom well plate. Centrifuge at 400 × *g* for 3 min at 4°C. Resuspend the cells in 200 μL of PBS. Centrifuge again and remove supernatant.
26. Dilute Zombie Aqua Fixable Viability Kit (BioLegend) at a dilution of 1:200 in PBS.
27. Resuspend the cells with 100 μL Zombie Aqua Fixable Viability Kit solution and incubate at room temperature (15°C–25°C) for 15 min. The plate should be covered with aluminum foil.
28. Centrifuge at 400 × *g* for 3 min at 4°C and discard the supernatant.
29. Dilute anti-mouse CD16/32 antibody (Fc block) at a dilution of 1:200 with 5% FACS buffer.
30. Resuspend the cells with 100 μL Fc block solution and incubate the cells on ice for 5 min.
31. During incubation, make a master mix solution of indicated antibodies (Table 1) in Eppendorf tubes or 15 mL conical tubes, depending on the total volume required.
32. After incubation with Fc block, centrifuge at 400 × *g* for 3 min at 4°C and remove the supernatant.
33. Add 100 μL of antibody master mix and incubate the cells on ice, in the dark, for 25 min.

**CRITICAL:** For the ILC panel, all antibodies except for two antibodies (GATA3 and RORγt) should be added at this step. Anti-GATA3 and -RORγt antibodies will be used in step 39.

34. Centrifuge at 400 × *g* for 3 min at 4°C and discard the supernatant.
35. Wash the cells with 200 μL of 5% FACS buffer.
36. Centrifuge at 400 × *g* for 3 min at 4°C and discard the supernatant.
37. Repeat steps 35 and 36.
38. Resuspend the cells with 200 μL of 5% FACS buffer. Keep cells stained for keratinocyte and myeloid cell panels on ice until data collection.
  ***Optional:*** Cells stained for keratinocyte panel and myeloid panels may be fixed with FluoroFix™ Buffer after step 37 if immediate data collection is not possible.
  a. Fix cells by adding 200 μL of FluoroFix™ Buffer. Incubate the cells at room temperature in the dark for 30 min.
  b. Centrifuge at 400 × *g* for 3 min at 4°C and discard the supernatant.
  c. Resuspend the cells with 200 μL of 5% FACS buffer.
  d. Centrifuge at 400 × *g* for 3 min at 4°C and discard the supernatant.
  e. Repeat steps (c) and (d).
  f. Resuspend the cells with 200 μL of 5% FACS buffer. Keep cells on ice until data collection.
39. To detect transcription factors in the ILC panel, use eBioscience™ Foxp3/Transcription Factor Staining Buffer Set.
  a. Fix cells by adding 200 μL of 1× Foxp3 Fixation/Permeabilization working solution. Incubate the cells at room temperature, in the dark, for 30 min.
  b. Centrifuge at 400 × *g* for 3 min at 4°C and discard the supernatant.
  c. Resuspend the cells with 200 μL of 1× Permeabilization Buffer working solution.
  d. Centrifuge at 400 × *g* for 3 min at 4°C and discard the supernatant.
  e. During the centrifuge step, prepare 1× Permeabilization Buffer working solution with 2% normal rat serum. Prepare 100 μL/sample.
  f. Resuspend cells with 100 μL of 1× Permeabilization Buffer working solution with 2% normal rat serum and incubate the cells at room temperature, in the dark, for 15 min.
  g. Prepare the antibody master mix for transcription factors GATA3 and RORγt in 1× Permeabilization Buffer working solution with 2% normal rat serum to achieve indicated final dilutions (Table 5).

**Table 5:** Transcription factor staining

Fluorophore	Marker	Clone	Final dilution
AF647	GATA3	L50-823	1/20
PE	RORγt	Q31-378	1/50

Make an antibody master mix immediately before the staining step.
  h. Add antibody master mix to each sample and incubate the cells at 4°C, in the dark, overnight (approximately 12 h).
  i. Centrifuge at 400 × *g* for 3 min at 4°C and discard the supernatant.
  j. Wash the cells with 200 μL 1× Permeabilization Buffer working solution.
  k. Centrifuge at 400 × *g* for 3 min at 4°C and discard the supernatant.
  l. Repeat steps (j) and (k).
  m. Resuspend the cells with 200 μL 1× Permeabilization Buffer working solution. Keep cells on ice until data collection.

### Data collection

**Timing: 2–3 h**

40. Collect data with LSR ll or LSR Fortessa (BD Biosciences) and analyze by using FlowJo software (FlowJo, LLC). Before acquiring samples, set appropriate PMT voltage and compensation by using compensation beads (Cat# 552845, BD) and a zombie aqua single-stained sample. Note that the compensation beads utilized here are specific for rat and hamster IgGs. If the antibody panels are modified to include antibodies generated in other host species, consider using other compensation bead products. Adjust compensation using the same set of antibodies from each panel.

## Expected outcomes

**Figure 2:**
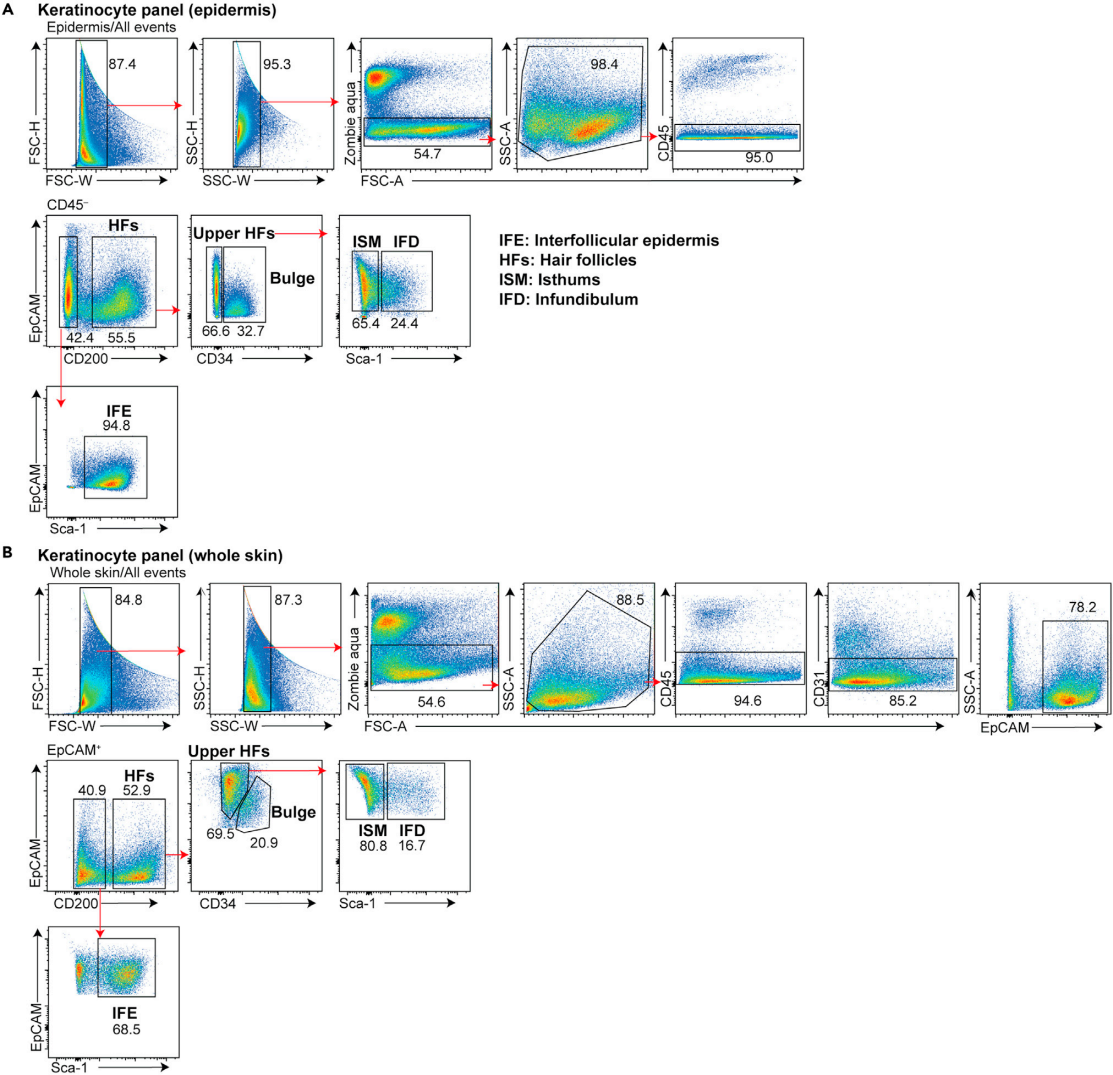
Gating strategy for keratinocytes (A) The gating strategy of interfollicular (EpCAM^+^ CD200^–^ Sca-1^+^) and HF keratinocytes (EpCAM^+^ CD200^+^) in epidermal cell suspensions. The HFs are first separated into the bulge (CD200^+^ CD34^+^) and upper HFs (CD200^+^ CD34^–^). Upper HFs are then separated into the infundibulum (EpCAM^+^ CD200^+^ CD34^–^ Sca-1^+^) and the isthmus (EpCAM^+^ CD200^+^ CD34^–^ Sca-1^–^). (B) The gating strategy of interfollicular (EpCAM^+^ CD200^–^ Sca-1^+^) and HF keratinocytes (EpCAM^+^ CD200^+^) in whole skin cell suspensions. The gating strategy is the same as in epidermal cell suspensions except for the exclusion of CD31^+^ vascular endothelial cells after gating for CD45^–^ cells.

Keratinocyte panel: Doublets are gated out first by FSC-H versus FSC-W and then by SSC-H versus SSC-W gates. Dead cells are subsequently excluded with Zombie Aqua staining. Because keratinocytes continuously turn over, it is normal to see up to 50% of epidermal cells to be positive for Zombie Aqua. Then, a broad gate for SSC-A versus FSC-A is made because the keratinocyte profile in this plot is broad. SSC-A^low^ FSC-A^low^ events represent debris and are excluded. CD45^–^ cells are gated to include all epidermal keratinocytes and to exclude immune cells. Further plotting for EpCAM and CD200 enables the distinction of keratinocytes from the interfollicular epidermis or HFs. CD200^+^ cells contain all HF subsets. The vast majority of CD200^–^ cells are Sca-1^+^ keratinocytes from the interfollicular epidermis. Among CD200^+^ HF cells, the CD34^+^ population represents the bulge cells (the stem cell area), and CD34^–^ cells represent the upper HFs, which are further divided into EpCAM^+^ Sca1^+^ infundibulum (the HF opening) and EpCAM^+^ Sca1^–^ isthmus (narrowing portion of the HFs below the infundibulum and above the bulge) (Sakamoto et al., 2021). This staining strategy for keratinocyte subsets is useful for analysis in non-inflamed skin (Figure 2A). In inflamed skin, the epidermal cell suspensions may be challenging to prepare. Thus, the same antibody panel may be applied to whole skin suspensions. In this case, while CD34^+^ bulge population is discernible, its separation is not as good as those observed in epidermal cell suspensions. Additionally, because CD31^+^ endothelial cells express CD200 (Ko et al., 2009), they need to be excluded by including a CD31 antibody in the antibody panel (Figure 2B).

**Figure 3:**
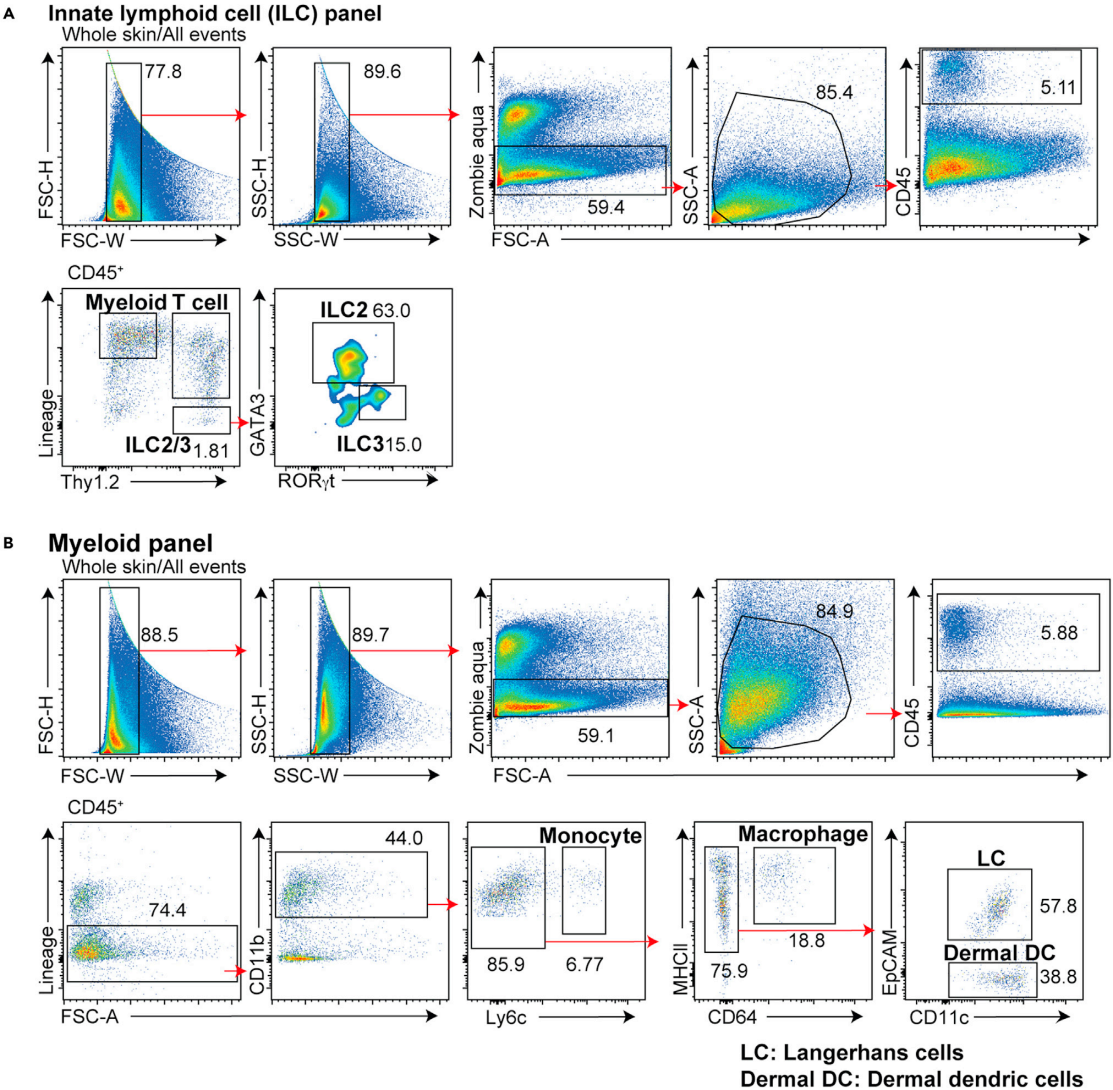
Gating strategy for skin ILCs and myeloid cells (A) The gating strategy for ILCs. Lineage markers include CD3ε, CD5, CD19, CD11b, Cd11c, FcεRIα, NK1.1 and CD2. Lin^+^ Thy1^–^ cells are myeloid cells. The majority of Lin^+^ Thy1^+^ cells are T cells. Lineage^–^ Thy1^+^ cells are identified as a mixture of ILC2s and ILC3s. From this gate, GATA3^high^ cells are ILC2 and GATA3^low^ RORγt^high^ cells are ILC3s. (B) The gating strategy of skin myeloid cells. Lineage markers include CD3ε, CD19, NK1.1 and Siglec-F. Gate for CD45^+^ lineage^–^ (Siglec-F^–^, CD3ε^–^, CD19^–^ and NK1.1^–^) to exclude eosinophils, T cells, B cells and NK cells. Lineage^–^ CD11b^+^ Ly6c^+^ cells are identified as monocytes. From the CD11b^+^ Ly6c^–^ gate, macrophages (EpCAM^–^ CD64^+^), Langerhans cells (CD64^–^ EpCAM^+^ MHC II^+^) and dermal dendritic cells (CD64^–^ EpCAM^–^ MHC II^+^ CD11c^+^) are identified.

ILC panel: Doublets are gated out first by FSC-H versus FSC-W and then by SSC-H versus SSC-W gates. Dead cells are subsequently excluded with Zombie Aqua staining. SSC-A versus FSC-A gate is used to exclude cell debris (SSC-A^low^ FSC-A^low^). Because fixation and permeabilization steps induce shrinkage of the cells, it is normal to see relatively lower SSC-A and FSC-A profiles as compared to those observed for unfixed cells. Immune cells are then identified via CD45^+^ versus FSC-A plot. CD45^+^ lineage^–^ (CD3ε^–^, CD11b^–^, CD11c^–^, CD5^–^, CD19^–^, FcεRIα^–^, NK1.1^–^ and CD2^–^) Thy1.2^+^ cells are a mixture of ILC2s and ILC3s (Sakamoto et al.). Lineage^+^ Thy1.2^–^ cells are myeloid cells and lineage^+^Thy1.2^+^ cells represent T cells. GATA3^high^ cells are ILC2 and GATA3^low^ RORγt^high^ cells are ILC3s. If ILC1 analysis is desired, then NK1.1 and CD2 can be independently stained. In this case, Lineage^–^ Thy1.2^+^ CD2^+^ population likely represent ILC1s (Figure 3A) (Sakamoto et al., 2021). It should be noted that skin ILC1s have not been definitively studied and the surface markers that they express in the skin (e.g., NK1.1) have yet to be determined.

Myeloid panel: Doublets, dead cells and debris are excluded, and immune cells are identified as described above. Gate for CD45^+^ lineage^–^ (Siglec-F^–^, CD3ε^–^, CD19^–^ and NK1.1^–^) to exclude eosinophils, T cells, B cells and NK cells. CD11b^+^ myeloid cells are gated and then Ly6c^+^ cells are identified as monocytes. From the CD11b^+^ Ly6c^–^ gate, EpCAM^–^ CD64^+^ cells are macrophages, CD64^–^ EpCAM^+^ MHC II^+^ cells are Langerhans cells, and CD64^–^ EpCAM^–^ MHC II^+^ cells are dermal CD11b^+^ CD11c^+^ dendritic cells (Figure 3B) (Nagao et al., 2009; Tamoutounour et al., 2013).

## Quantification and statistical analysis

FlowJo (FlowJo, LLC https://www.flowjo.com/solutions/flowjo) was used for data analysis. To avoid potential data variability among experiments or among different experimentalists, total collected events were fixed among samples. The total number of cells that are acquired can be optimized depending on the abundance of the specific skin cell subsets (Table 6).

**Table 6:** Total collected events and expected percentage of live cells and CD45^+^ cells in C57BL/6 mouse skin

	Keratinocyte panel	ILC panel	Myeloid panel
Total collected events	200,000	300,000–1,000,000	300,000
Live cells	50–60%	50–60%	50–60%
CD45^+^ cells	3–5%	3–5%	3–5%

Because of the low cell numbers of ILCs, we recommend collecting 1 million events if analyzing the skin during steady state. If inflammatory models are analyzed, the number of events to be acquired should be determined for each model.

## Limitations

Adult mice develop spontaneous anagen (growth phase of the hair cycle) patches after 12 weeks of age (Müller-Röver et al., 2001). While this is only rarely observed in the chest area, it is commonly observed in back skin. Epidermal cell suspensions may be challenging to prepare from such areas. Whole skin processing of anagen patches require proper mincing of the tissues. Trauma or dermatitis may also induce anagen patches. If unmanipulated mice show extensive anagen patches, researchers should assess the skin surface for the possibility of trauma or inflammation. Such mice may have to be excluded from analysis.

Antibody panels must be optimized for each flow cytometer. Signals can be prominently affected by the types of lasers, voltage settings, and the combination of filters that flow cytometers are equipped with. We recommend that the antibody panels are optimized with single-stained samples as well as “fluorescence minus one”, or FMO, for all antibodies.

We have included NK1.1 in our lineage channel to exclude NK cells. However, exclusion of NK cells in non-C57BL/6 strains such as Balb/C mice, in which NK1.1 is not expressed (Carlyle et al., 2006), may require the use of other NK cell markers such as CD49b that is detected by the antibody DX5 (Arase et al., 2001).

## Troubleshooting

### Problem 1

Experimental mice display prominent anagen patches (step 2).

### Potential solution

Make sure that all utilized mice are of the same age and at most 1 week apart to ensure the hair cycle is comparable. We recommend experimental mice to be 8.5–12 weeks of age at the time of harvest to avoid anagen skin. If later ages need to be analyzed, and if hair cycle is not the focus of the experiment, consider excluding anagen patches when harvesting skin. Barbering and fighting can traumatize the skin and trigger anagen, potentially leading to incomparable skin conditions between experimental mice. If trauma is an issue, house mice in individual cages 1 or 2 weeks prior to harvest.

### Problem 2

Epidermal cells do not detach well from the dermis after trypsin treatment (step 3).

### Potential solution

Make sure that subcutaneous tissue is removed completely. Remaining subcutaneous tissue may interfere with enzymatic digestion. One may test effective digestion by scraping off the epidermis in one of the samples at the end of the 45 min incubation with epidermal digestion solution. If epidermal detachment is poor, further incubate samples for additional 5–10 min. Additional incubation for over 10 min is not recommended because longer incubation could potentially lead to the digestion of cell surface markers. One common mistake is including FBS in the epidermal digestion solution. Make sure digestion solutions do not contain FBS because it will interfere with enzymatic digestion. If none of the above improve tissue dissociation, make a new batch of epidermal dissociation solution. Epidermal cells will not detach well in inflamed skin. If an inflammatory skin model is being studied, consider using whole skin suspensions.

### Problem 3

Cells are not retrieved from whole skin digestion (step 24).

### Potential solution

Thorough mincing is critical in generating single cell suspensions from whole skin samples. If cells still cannot be retrieved in sufficient numbers, make sure the skin samples are minced well next time. One common mistake is including FBS in the whole skin digestion solution, which will interfere with enzymatic digestion. If none of the above improve tissue dissociation, try a different lot of Liberase T-Flex.

### Problem 4

Up to 50% of epidermal and whole skin cell suspensions may be positive for the viability dye. However, in certain instances, a higher percentage of cells could be non-viable (step 40).

### Potential solution

Non-immune cells, in particular keratinocytes, may continuously undergo cell death even if the cell suspensions are kept on ice. Thus, delays in cell processing may lead to increased percentages of dead cells. After the generation of epidermal and whole skin cell suspensions, samples should be immediately stained, and data must be collected in a timely manner. If a timely data collection is not possible, immediately fix the cells after antibody staining is done.

### Problem 5

No positive staining of some markers by flow cytometry (step 40).

### Potential solution

Prior to the acquisition, ensure proper flow cytometer settings. Acquire a small amount of full-stained samples and check if all targeted populations show up.

Ensure that antibodies have been correctly stored and are not expired.

Ensure that antibodies were added and used at the suitable concentration. If one is unsure if a particular antibody was added, go back to the staining step (33). However, cells will be lost during this extra washing step.

Pair the low expressing antigens with bright fluorochromes such as PE or APC next time.

If none of the above work, try another antibody clone.

## Resource availability

### Lead contact

Further information and requests for resources and reagents should be directed to and will be fulfilled by the lead contact, Keisuke Nagao (keisuke.nagao@nih.gov).

### Materials availability

No new materials were generated in this protocol.

## Data Availability

This study did not generate or analyze any datasets.
